# Supplementary material for: Relationships between hemoglobin levels at admission and adverse maternal and perinatal outcomes in patients with preeclampsia
Source: PLoS One. 2025 Oct 16;20(10):e0335079. doi: 10.1371/journal.pone.0335079 (PMC12530512; doi:10.1371/journal.pone.0335079)
Supplement: Attachment — Submitted filename: Response to Reviewers.docx [file pone.0335079.s001.docx]

PONE-D-24-46963

July 24, 2025

Dear Dr. He,

Thank you for submitting your manuscript to PLOS ONE. After careful consideration, we feel that it has merit but does not fully meet PLOS ONE’s publication criteria as it currently stands. Therefore, we invite you to submit a revised version of the manuscript that addresses the points raised during the review process.

Please submit your revised manuscript by Sep 06 2025 11:59PM. If you will need more time than this to complete your revisions, please reply to this message or contact the journal office at plosone@plos.org. When you're ready to submit your revision, log on to https://www.editorialmanager.com/pone/ and select the 'Submissions Needing Revision' folder to locate your manuscript file.

Please include the following items when submitting your revised manuscript:

A rebuttal letter that responds to each point raised by the academic editor and reviewer(s). You should upload this letter as a separate file labeled 'Response to Reviewers'.

A marked-up copy of your manuscript that highlights changes made to the original version. You should upload this as a separate file labeled 'Revised Manuscript with Track Changes'.

An unmarked version of your revised paper without tracked changes. You should upload this as a separate file labeled 'Manuscript'.

If you would like to make changes to your financial disclosure, please include your updated statement in your cover letter. Guidelines for resubmitting your figure files are available below the reviewer comments at the end of this letter.

If applicable, we recommend that you deposit your laboratory protocols in protocols.io to enhance the reproducibility of your results. Protocols.io assigns your protocol its own identifier (DOI) so that it can be cited independently in the future. For instructions see: https://journals.plos.org/plosone/s/submission-guidelines#loc-laboratory-protocols. Additionally, PLOS ONE offers an option for publishing peer-reviewed Lab Protocol articles, which describe protocols hosted on protocols.io. Read more information on sharing protocols at https://plos.org/protocols?utm_medium=editorial-email&utm_source=authorletters&utm_campaign=protocols.

We look forward to receiving your revised manuscript.

Kind regards,

Ali Cetin

Academic Editor

PLOS ONE

**Response to Reviewers’ Comments**

Manuscript ID: PONE-D-24-46963

Title: Relationships between hemoglobin levels at admission and adverse maternal and perinatal outcomes in preeclampsia patients: A retrospective study

Journal: PLOS ONE

Corresponding Author: Tongqiang He, E-mail: xbfymicu@163.com

Dear Dr. Ali Cetin,

We sincerely appreciate the reviewer’s constructive comments and the opportunity to revise our manuscript. We have revised the manuscript and answered reviewer's questions/comments with point by point hereunder according to the requirements in your letter.

To easily distinguish my answers from reviewer’s questions/comments, we highlighted all our answers in blue while keeping your letter and reviewer’s questions/comments in black in the Response letter.

Thank you again for your time and consideration.

Looking forward to hearing from you.

Best regards,

Tongqiang He

Department of Obstetrics and Gynecology Intensive Care Unit, Northwest Women's and Children's Hospital

Email: xbfymicu@163.com

Journal Requirements:

When submitting your revision, we need you to address these additional requirements.

1. Please ensure that your manuscript meets PLOS ONE's style requirements, including those for file naming. The PLOS ONE style templates can be found at

https://journals.plos.org/plosone/s/file?id=wjVg/PLOSOne_formatting_sample_main_body.pdf and

https://journals.plos.org/plosone/s/file?id=ba62/PLOSOne_formatting_sample_title_authors_affiliations.pdf

**Reply:** Thank you very much for your patient guidance. We have carefully checked the revised manuscript again to ensure that the format and file meet PLOS ONE's style requirements.

2. We note that the grant information you provided in the ‘Funding Information’ and ‘Financial Disclosure’ sections do not match.

When you resubmit, please ensure that you provide the correct grant numbers for the awards you received for your study in the ‘Funding Information’ section.

**Reply:** We apologize for the discrepancy between the ‘Funding Information’ and ‘Financial Disclosure’ sections. The funding information is hereby confirmed as follows: This project is supported by the Key Research and Development Program of Shaanxi China (Program number 2024SF-YBXM-230). The funders have no role in study design, data collection and analysis, the decision to publish, or the preparation of the manuscript. No author receives a salary from any of the funders. The Key Research and Development Program of Shaanxi is a sub-organization of Shaanxi Provincial Science and Technology Department. This inconsistency may be due to an oversight on our part when submitting the manuscript. We have corrected our funding information in the cover letter.

3. Please note that funding information should not appear in the Acknowledgments section or other areas of your manuscript. We will only publish funding information present in the Funding Statement section of the online submission form. Please remove any funding-related text from the manuscript.

**Reply:** Thank you for reminding us. We have removed the relevant financial information from my manuscript.

4. We note that your Data Availability Statement is currently as follows:

“All relevant data are within the manuscript and its Supporting Information files.”

Please confirm at this time whether or not your submission contains all raw data required to replicate the results of your study. Authors must share the “minimal data set” for their submission. PLOS defines the minimal data set to consist of the data required to replicate all study findings reported in the article, as well as related metadata and methods (https://journals.plos.org/plosone/s/data-availability#loc-minimal-data-set-definition).

For example, authors should submit the following data:

- The values behind the means, standard deviations and other measures reported;

- The values used to build graphs;

- The points extracted from images for analysis.

Authors do not need to submit their entire data set if only a portion of the data was used in the reported study.

If your submission does not contain these data, please either upload them as Supporting Information files or deposit them to a stable, public repository and provide us with the relevant URLs, DOIs, or accession numbers. For a list of recommended repositories, please see https://journals.plos.org/plosone/s/recommended-repositories.

If there are ethical or legal restrictions on sharing a de-identified data set, please explain them in detail (e.g., data contain potentially sensitive information, data are owned by a third-party organization, etc.) and who has imposed them (e.g., an ethics committee). Please also provide contact information for a data access committee, ethics committee, or other institutional body to which data requests may be sent. If data are owned by a third party, please indicate how others may request data access.

**Reply:** Due to privacy and ethical restrictions, data cannot be shared publicly. However, researchers meeting the criteria for access to relevant data may obtain it from the Ethics Committee of Northwest Women's and Children's Hospital via email at xbfyyxll@163.com.

5. When completing the data availability statement of the submission form, you indicated that you will make your data available on acceptance. We strongly recommend all authors decide on a data sharing plan before acceptance, as the process can be lengthy and hold up publication timelines. Please note that, though access restrictions are acceptable now, your entire data will need to be made freely accessible if your manuscript is accepted for publication. This policy applies to all data except where public deposition would breach compliance with the protocol approved by your research ethics board. If you are unable to adhere to our open data policy, please kindly revise your statement to explain your reasoning and we will seek the editor's input on an exemption. Please be assured that, once you have provided your new statement, the assessment of your exemption will not hold up the peer review process.

**Reply:** I'm sorry, but due to restrictions imposed by the ethics committee of our hospital, and the fact that our current research involves this raw data. The raw data cannot be shared publicly. We will update our data availability statement in the revised manuscript.

6. Please include a separate caption for figure 1 in your manuscript.

**Reply:** We have added a separate caption for Figure 1 in the revised manuscript.

If the reviewer comments include a recommendation to cite specific previously published works, please review and evaluate these publications to determine whether they are relevant and should be cited. There is no requirement to cite these works unless the editor has indicated otherwise.

**Reply:** The reviewers did not request to cite specific published works.

Additional Editor Comments:

I have reviewed this retrospective study examining hemoglobin levels and adverse outcomes in preeclampsia patients, along with two peer reviewer reports. One reviewer recommends rejection with strong methodological concerns, while the other suggests minor revision with more optimistic assessment. After careful consideration, I find this manuscript addresses a clinically relevant question with adequate sample size but has significant limitations preventing publication in its current form.

**Reply:** We are very grateful for your valuable constructive comments on our manuscript and thank you for giving us the opportunity to revise it. We are deeply appreciative and have made detailed revisions based on your review comments.

The most critical deficiency is exclusive use of univariate analysis. Contemporary medical research requires multivariable adjustment for observational studies examining exposure-outcome associations. The absence of logistic regression controlling for confounders such as maternal age, BMI, gestational age, and preeclampsia severity renders all reported odds ratios unreliable. Without these adjustments, conclusions cannot be considered valid.

**Reply:** The limitation of our study is that we only performed a univariate binary logistic regression analysis. We appreciate you pointing out this limitation and giving us the opportunity to revise it. We reanalyzed some of the data, such as categorizing the number of previous deliveries into two groups based on nulliparous and multiparous. Additionally, we classified preeclampsia severity as either mild or severe. These results are presented in Table 1. To minimize the influence of confounding factors on the impact of anemia and high hemoglobin level on adverse pregnancy outcomes in preeclampsia patients, we included maternal age, pre-pregnancy BMI, gestational age at onset of preeclampsia and the presence of severe preeclampsia, nulliparas, and assisted reproduction as covariates in our binary logistic regression analysis. The results of the multivariate regression analysis indicate that specific adverse pregnancy outcomes are associated with hemoglobin levels. Compared with preeclampsia patients with normal hemoglobin levels, patients with anemia at admission were at a greater risk for postpartum hemorrhage (OR: 3.800; 95% CI: 1.677–8.610) and cardiac dysfunction (OR: 2.860; 95% CI: 0.979–8.356). Moreover, patients with high hemoglobin levels at admission were at an increased risk for developing HELLP syndrome (OR: 2.503; 95% CI: 1.198–5.229). With respect to perinatal outcomes, we found that preeclampsia patients with high hemoglobin levels were at an increased risk of SGA and NICU admission, whereas patients with anemia were at a reduced risk. In addition, preeclampsia patients with high hemoglobin levels were at an increased risk of neonatal asphyxia (OR: 2.046; 95% CI: 1.107–3.784). To present the research results more intuitively, we used a forest plot to visually display the OR (95% CI) of the association between anemia and high hemoglobin levels with adverse pregnancy outcomes in Fig 2.

| **Table 1. Baseline clinical characteristics of the study population** | | | | | |
| --- | --- | --- | --- | --- | --- |
| **Characteristics** | **Anemia group** | **Normal hemoglobin level group** | **High hemoglobin level group** | **Statistics** | **P value** |
| N | 395 | 877 | 443 | / | / |
| Age, years | 31.17±4.63 | 31.41±4.43 | 31.38±4.18 | *F*=*0.419* | 0.658 |
| Pre-pregnancy BMI, kg/m^2^ | 23.84±4.35 | 24.01±4.24 | 23.60±3.84 | *F*=*1.450* | 0.235 |
| Gestational age at the onset of preeclampsia, weeks | 33.89±4.24 | 33.97±4.23 | 34.08±4.02 | *F*=*0.234* | 0.791 |
| Severe preeclampsia, n(%) | 359(90.9) | 781(89.1) | 414(93.5) | *χ^2^*=*6.744* | 0.034 |
| Nulliparas, n(%) | 222(56.2) | 534(60.9) | 275(62.1) | *χ^2^*=*3.452* | 0.178 |
| Assisted reproduction, n(%) | 37(9.4) | 103(11.7) | 59(13.3) | *χ^2^*=*3.213* | 0.201 |
| Gestational age at termination of pregnancy, weeks | 34.98±3.64 | 35.01±3.60 | 34.88±3.55 | *F*=0.184 | 0.832 |
| Birth weight of newborns, g |  |  |  |  |  |

Footnote: BMI, body mass index.

**Fig 2. Odds ratios for adverse pregnancy outcomes associated with anemia and high hemoglobin levels.**

Abbreviations: OR, odds ratio; HELLP syndrome, eclampsia and hemolysis, elevated liver enzymes, and thrombocytopenia syndrome; IUFD, intrauterine fetal death; SGA, small for gestational age.

This manuscript exhibits concerning similarity to previously published literature, particularly in introduction and discussion sections. Multiple passages use language closely mirroring existing publications, suggesting inadequate paraphrasing or potential plagiarism. Most journals require similarity indices below 15%. Additionally, several sections display characteristics consistent with AI-generated writing, including unnaturally polished prose and formulaic sentence structures that lack authentic scientific voice.

**Reply:** When we first wrote this paper, we borrowed sentence structures from related articles and used basic translation software. Although we carefully revised the paper, some sentences still sound awkward because English is not our native language. We reduced the repetition rate in the revised manuscript. To improve its quality, we contacted a professional English editing company to make the English more accurate and correct basic errors. Proofreading certificate as follows:

The single-center retrospective design from a tertiary referral hospital introduces substantial selection bias. While authors acknowledge receiving "critically ill pregnant women," they inadequately address how this population differs from typical preeclampsia patients, limiting generalizability. Multiple comparison testing without statistical correction inflates false-positive risk, and outcome definitions like "early-stage heart failure" lack specificity.

**Reply:** In this study, 90.6% of pregnant women developed severe preeclampsia, a proportion that exceeds the 65.5% rate of severe preeclampsia observed among preeclampsia patients in China(1). We analyzed severe preeclampsia, maternal age, pre-pregnancy BMI, gestational age at onset of preeclampsia and the presence of nulliparas, and assisted reproduction as covariates in the multivariate binary logistic regression analysis, which to some extent reduced the impact of the severity of preeclampsia on the results. Despite the potential for selection bias, the study's substantial sample size offers a valuable opportunity to address some clinical concerns with hemoglobin levels, particularly in tertiary hospitals with a high proportion of patients experiencing severe preeclampsia. Future multicenter studies with larger samples are necessary to further investigate the impact of hemoglobin levels on adverse pregnancy outcomes in patients with preeclampsia.

In Chinese obstetrics and gynecology textbooks, early heart failure is a clinical concept with diagnostic criteria that include: 1. Chest tightness, palpitations, and shortness of breath after minimal activity.2. Resting heart rate greater than 110 beats per minute and respiratory rate greater than 20 breaths per minute.3. Frequent need to sit up to breathe or go to a window for fresh air at night due to chest tightness.4. Persistent fine crackles in the lungs that do not disappear after coughing. The diagnosis of early heart failure can be confusing to obstetricians outside China, so we have combined early heart failure and heart failure into cardiac dysfunction.

Data presentation has notable deficiencies including missing confidence intervals in Table 2, inadequate statistical methods description, and no power calculation. The temporal relationship between hemoglobin measurement and adverse outcomes is unclear, raising causation questions.

**Reply:** The incidence of adverse pregnancy outcomes in preeclampsia patients with different hemoglobin levels has been meticulously delineated in Table 2. In the revised manuscript, we have incorporated the 95% confidence intervals for the incidence rates of various adverse pregnancy outcomes, chi-square values, and post-hoc power analyses. However, due to the low incidence rates or minimal intergroup differences in some adverse pregnancy outcomes, the post-hoc power was less than 80%. However, we should also note the limitations of post-hoc power. Insignificant results mathematically guarantee low post-hoc power, which does not provide any additional information beyond the p-value itself. In addition, we included relevant covariates in the subsequent multivariate logistic regression, which to a certain extent increased the credibility of the results. Although we can only describe the association between different hemoglobin levels and adverse pregnancy outcomes due to the nature of the observational study, our findings have implications for the clinical management of preeclampsia patients, particularly those with severe preeclampsia. We should focus on different aspects in the clinical management of patients with preeclampsia at different hemoglobin levels. Some adverse outcomes may not be caused by abnormal hemoglobin levels due to the complexity of the pathogenesis of preeclampsia. The specific mechanisms by which hemoglobin influence pregnancy outcomes in preeclampsia patients require further investigation.

| **Table 2. Incidence of adverse maternal and perinatal outcomes in preeclampsia patients with three hemoglobin levels** | | | | | | | | | | | |
| --- | --- | --- | --- | --- | --- | --- | --- | --- | --- | --- | --- |
| Variables | Anemia | |  | Normal hemoglobin level | |  | High hemoglobin level | | *χ^2^* | P value | Post hoc power,% |
|  | n | Rate,%(95% CI) |  | n | Rate,%(95% CI) |  | n | Rate,%(95% CI) |  |  |  |
| Maternal |  |  |  |  |  |  |  |  |  |  |  |
| Cardiac dysfunction | 8 | 2.0(0.9,4.0) |  | 6 | 0.7(0.3,1.5) |  | 8 | 1.8(0.8,3.5) | 5.159 | 0.076 | 52.4 |
| Eclampsia | 2 | 0.5(0.1,1.8) |  | 9 | 1.0(0.5,1.9) |  | 11 | 2.5(1.2,4.4) | 7.376 | 0.025 | 67.6 |
| Postpartum hemorrhage | 15 | 3.8(2.1,6.2) |  | 11 | 1.3(0.6,2.2) |  | 4 | 0.9(0.2,2.3) | 12.738 | 0.002 | 90.1 |
| HELLP syndrome | 9 | 2.3(1.0,4.3) |  | 13 | 1.5(0.8,2.5) |  | 17 | 3.8(2.3,6.1) | 7.346 | 0.025 | 67.5 |
| Placental abruption | 23 | 5.8(3.7,8.6) |  | 51 | 5.8(4.4,7.6) |  | 23 | 5.2(3.3,7.7) | 0.241 | 0.886 | 6.8 |
| Cesarean delivery | 362 | 91.6(88.5,94.2) |  | 779 | 88.8(86.6,90.8) |  | 401 | 90.5(87.4,93.1) | 2.63 | 0.268 | 28.7 |
| Perinatal |  |  |  |  |  |  |  |  |  |  |  |
| Premature delivery | 229 | 58(52.9,62.9) |  | 506 | 57.7(54.3,61.0) |  | 278 | 62.8(58.1,67.3) | 3.367 | 0.186 | 35.7 |
| IUFD | 15 | 3.8(2.1,6.2) |  | 14 | 1.6(0.9,2.7) |  | 6 | 1.4(0.5,2.9) | 8.008 | 0.018 | 70.9 |
| SGA | 56 | 14.2(10.9,18.0) |  | 154 | 17.6(15.1,20.2) |  | 97 | 21.9(18.1,26.0) | 8.608 | 0.014 | 75.1 |
| Neonatal asphyxia | 14 | 3.5(2.0,5.9) |  | 24 | 2.7(1.8,4.0) |  | 23 | 5.2(3.3,7.7) | 5.173 | 0.075 | 51.6 |
| NICU admission | 158 | 40(35.1,45.0) |  | 396 | 45.2(41.8,48.5) |  | 231 | 52.1(47.4,56.9) | 12.684 | 0.002 | 90.1 |

Footnote: HELLP syndrome, eclampsia and hemolysis, elevated liver enzymes, and thrombocytopenia syndrome; IUFD, intrauterine fetal death; SGA, small for gestational age; NICU, neonatal intensive care unit.

The manuscript requires professional English editing due to numerous grammatical errors and awkward phrasing throughout. Important variables like iron supplementation, dietary factors, and severity markers are missing from analysis.

**Reply:** A native English-speaking proofreading company was commissioned to correct grammatical and stylistic errors in the revised manuscript. A notable limitation of our research database is the absence of data concerning patients' dietary habits and iron intake prior to hospitalization, which restricts the comprehensiveness of the available information. However, the present study focuses on observing the impact of hemoglobin levels on adverse pregnancy outcomes, rather than on the risk factors for anemia or high hemoglobin levels. While dietary habits and iron supplement intake have been demonstrated to have a significant impact on hemoglobin levels, their direct influence on adverse pregnancy outcomes in patients with preeclampsia is relatively small. In subsequent studies, we will incorporate prenatal dietary habits and iron supplement intake into our analysis. We added preeclampsia severity as a covariate in the revised manuscript and analyzed it in the multivariate regression analysis, thereby reducing the influence of preeclampsia severity on the relationship between hemoglobin levels and adverse pregnancy outcomes. We will further explore the impact of different severity markers of preeclampsia on pregnancy outcomes in future studies.

For successful revision, authors must conduct multivariable logistic regression analysis, eliminate AI-generated content and rewrite affected sections authentically, reduce text similarity through original writing, clarify outcome definitions and measurement timing, and obtain professional language editing. Additional improvements should include subgroup analysis by preeclampsia severity and enhanced discussion of limitations.

**Reply:** Thanks for your suggestion. To enhance the reliability of the study results, we conducted a multivariate logistic regression analysis. The manuscript underwent a thorough revision and professional editing process in English. Although most adverse pregnancy outcomes in our study occurred after hospitalization, we have to acknowledge that some adverse outcomes, such as small-for-gestational-age infants, were present at the time of admission. This is a limitation of retrospective cross-sectional studies. Additionally, 90.6% of the patients in our study were diagnosed with severe preeclampsia. In the multivariate analysis model, severe preeclampsia was included as a covariate to control for its confounding effects, and this was discussed in the limitations section.

Weighing contrasting reviewer assessments, I recognize this manuscript's potential for meaningful contribution to obstetric literature. The substantial sample size and comprehensive outcomes provide solid foundation for valuable research.

However, fundamental statistical limitations and originality concerns require substantial attention. I recommend an A to Z revision with clear expectation that addressing methodological issues, particularly implementing proper multivariable analysis and ensuring complete content originality, will be essential for meeting publication standards. Authors should view this as an opportunity to significantly strengthen both scientific rigor and clinical impact of their important research.

**Reply:** We would like to express our gratitude once again for the opportunity to revise our manuscript and for your constructive comments. A multivariate regression analysis was conducted in accordance with the review comments, and the results indicate that anemia and elevated hemoglobin levels remain associated with adverse pregnancy outcomes. These findings underscore the significance of hemoglobin levels in patients with preeclampsia. A thorough revision of the manuscript was conducted, and the services of a professional native English editor were enlisted to enhance its quality. We hope the revised manuscript could be acceptable for you.

[Note: HTML markup is below. Please do not edit.]

Reviewers' comments:

Reviewer's Responses to Questions

Comments to the Author

1. Is the manuscript technically sound, and do the data support the conclusions?

The manuscript must describe a technically sound piece of scientific research with data that supports the conclusions. Experiments must have been conducted rigorously, with appropriate controls, replication, and sample sizes. The conclusions must be drawn appropriately based on the data presented.

Reviewer #1: Yes

Reviewer #2: Yes

2. Has the statistical analysis been performed appropriately and rigorously?

Reviewer #1: Yes

Reviewer #2: Yes

3. Have the authors made all data underlying the findings in their manuscript fully available?

The PLOS Data policy requires authors to make all data underlying the findings described in their manuscript fully available without restriction, with rare exception (please refer to the Data Availability Statement in the manuscript PDF file). The data should be provided as part of the manuscript or its supporting information, or deposited to a public repository. For example, in addition to summary statistics, the data points behind means, medians and variance measures should be available. If there are restrictions on publicly sharing data—e.g. participant privacy or use of data from a third party—those must be specified.

Reviewer #1: Yes

Reviewer #2: Yes

4. Is the manuscript presented in an intelligible fashion and written in standard English?

PLOS ONE does not copyedit accepted manuscripts, so the language in submitted articles must be clear, correct, and unambiguous. Any typographical or grammatical errors should be corrected at revision, so please note any specific errors here.

Reviewer #1: Yes

Reviewer #2: Yes

5. Review Comments to the Author

Please use the space provided to explain your answers to the questions above. You may also include additional comments for the author, including concerns about dual publication, research ethics, or publication ethics. (Please upload your review as an attachment if it exceeds 20,000 characters)

Reviewer #1: The authors in this retrospective study show that 3rd trimester maternal hemoglobin levels from PE patients are correlated with adverse maternal and perinatal outcomes of PE pregnancies. Based on their analysis, authors conclude that patients with PE and high hemoglobin levels are prone to develop severe complications like eclampsia, HELP syndrome. Authors also found that the birth weight of newborns across studied groups decreased with increases in maternal hemoglobin levels which is in accordance with other studies showing that mild anemia (100-109g/l) during pregnancy is associated with decreased risk of fetal growth restriction in pregnant women (Yuan Wei, Nutrients, 2023).

**Reply:** Thank you for taking the time to review our manuscript and provide valuable feedback. Our study found that maternal and perinatal adverse pregnancy outcomes were associated with hemoglobin levels after adjusting for variables such as maternal age, gestational age at onset of preeclampsia, and severity of preeclampsia, etc. These results suggest that hemoglobin levels have important clinical significance for patients with preeclampsia. For perinatal outcomes, preeclampsia patients with high hemoglobin levels had significantly increased risks of small for gestational age (SGA), neonatal asphyxia, and NICU admission, while anemic preeclampsia patients had reduced risks of SGA and NICU admission. This phenomenon suggests that the optimal hemoglobin threshold for preeclampsia patients may not align with that of normal pregnant women. However, due to the limited sample size, our study was unable to further explore the optimal hemoglobin threshold for preeclampsia patients. Further multicenter and larger-scale studies are necessary to ascertain the optimal hemoglobin levels for patients with preeclampsia.

Minor comments:

1. In Methods section ( page 6)- ...for the study, we defined gestational anemia as hemoglobine = or less 109g/l...

Although in Discussion, authors using term "mild anemia" , there is no such description or reference in Methods section.

Authors do not show how were the lowest levels of maternal hemoglobine among PE patients.

According to WHO, anemia in pregnant women is classified as mild, moderate and severe anemia.

It might be helpful to use this classification in data analysis.

**Reply:** The diagnostic criteria for mild anemia during pregnancy in China are hemoglobin concentrations of 100–109 g/L, moderate anemia is defined as hemoglobin concentrations of 70–99 g/L, and severe anemia is defined as hemoglobin concentrations <70 g/L. In our study, perinatal outcomes in preeclampsia patients with anemia included a reduced risk of SGA and NICU admission, which is inconsistent with findings in the general pregnant population that anemia increases the risk of SGA and NICU admission. This phenomenon suggests that the optimal hemoglobin concentration in preeclampsia patients may differ from that in normal pregnant women due to the pathophysiological changes associated with preeclampsia. Due to the low incidence of certain adverse pregnancy outcomes, our current study sample size is insufficient to support further hemoglobin grouping. This is an area we need to explore further in the future, requiring multicenter and larger-scale studies to determine the optimal hemoglobin levels for preeclampsia patients.

2. Discussion, p.13 - Anemia is associated.......with low birth weight and stillbirth.

This is a little confusing, because mild anemia (the term which authors using in the Discussion) is associated with decreased

risk of FGR and stillbirth.

**Reply:** In our study, the risk of SGA was reduced in preeclampsia cases with anemia. This may be because anemia in preeclampsia patients leads to decreased blood viscosity, which can improve uteroplacental blood flow to some extent. When anemia is not severe, the overall oxygen supply to the uterus and placenta may be better than in preeclampsia patients with elevated hemoglobin concentrations. However, as hemoglobin concentration decreases further, the blood's ability to carry oxygen decreases as well. This ultimately leads to a reduced oxygen supply to the uterus and placenta. This, in turn, increases the risk of fetal intrauterine death. However, our sample size was insufficient to support further exploration of the optimal hemoglobin level for the best perinatal outcomes in preeclampsia patients. This limitation of our study has been addressed in the limitations section of the manuscript. Further multicenter studies with larger sample sizes are needed to clarify the optimal hemoglobin concentration for different adverse pregnancy outcomes.

3. According to Methods section, the maternal hemoglobine samples from PE pregnancies were collected from 2018-2023.

It spanned Covid19 pandemic, that also has impacted pregnant women. Covid19 has also been linked to higher rates of preterm birth , high blood pressure, preeclampsia, prevalence of anemia in pregnancy.

If authors can shortly comment on this?

If e.g. in 2020-2021 was observed higher number of PE pregnancies when comparying with other years?

**Reply:** Thanks for your question. Although our data collection period spans from 2018 to 2023, due to the Chinese government's strict COVID-19 management policies, most Chinese people infected with COVID-19 within a very short period after the pandemic control measures were lifted in 2023. Prior to that, only a very small proportion of the Chinese population had contracted COVID-19. The same applies to pregnant women in Shaanxi Province, which means that the impact of the COVID-19 on our study was minimal. Therefore, we did not conduct a specific study on the effects of the COVID-19 virus on the outcomes of preeclampsia.

Reviewer #2: Though the article is well written it does not bring out anything which is not known. It is not clear on what basis the cut off of Hb > 13g% has been chosen. There is no comment on echocardiography findings in women and no additional haematological data except Hb

**Reply:** Thank you for taking the time to review our manuscript and provide valuable feedback. Although there have been many studies on the relationship between hemoglobin levels and adverse pregnancy outcomes, there are few studies on the relationship between hemoglobin levels and adverse pregnancy outcomes in preeclampsia. Our study preliminarily demonstrated that hemoglobin has important clinical significance for patients with preeclampsia. Our research also suggests that the impact of hemoglobin on pregnancy outcomes may differ from that in normal pregnant women due to the pathophysiological changes associated with preeclampsia.

The current definition standards for high hemoglobin concentrations are not uniform, with most studies defining high hemoglobin during pregnancy as greater than 130 g/L(2,3).

Our patients underwent echocardiography upon admission, and some patients reported diastolic dysfunction but normal systolic function, while most patients had no clinical manifestations. However, our diagnosis of cardiac dysfunction is primarily based on clinical indicators such as shortness of breath, auscultation of pulmonary crackles, and reduced oxygenation. Your question raises an excellent direction for our further research, and we will continue to explore the relationship between echocardiogram results and cardiac dysfunction in preeclampsia patients in future studies.

The primary objective of our study was to explore the relationship between hemoglobin levels and adverse pregnancy outcomes in preeclampsia. Other blood biochemical data had a minimal impact on our study, so we did not present the patients' biochemical data.

6. PLOS authors have the option to publish the peer review history of their article (what does this mean?). If published, this will include your full peer review and any attached files.

If you choose “no”, your identity will remain anonymous but your review may still be made public.

**Reply:** Yes, we agree to publish the peer review history of our article.

Do you want your identity to be public for this peer review? For information about this choice, including consent withdrawal, please see our Privacy Policy.

Reviewer #1: No

Reviewer #2: Yes: Anjoo Agarwal

[NOTE: If reviewer comments were submitted as an attachment file, they will be attached to this email and accessible via the submission site. Please log into your account, locate the manuscript record, and check for the action link "View Attachments". If this link does not appear, there are no attachment files.]

While revising your submission, please upload your figure files to the Preflight Analysis and Conversion Engine (PACE) digital diagnostic tool, https://pacev2.apexcovantage.com/. PACE helps ensure that figures meet PLOS requirements. To use PACE, you must first register as a user. Registration is free. Then, login and navigate to the UPLOAD tab, where you will find detailed instructions on how to use the tool. If you encounter any issues or have any questions when using PACE, please email PLOS at figures@plos.org. Please note that Supporting Information files do not need this step.

**Reply:** All our figures have been checked by PACE.

**References**

1.	Lin L, Huai J, Su R, Wang C, Li B, Yang H. Incidence and Clinical Risk Factors for Preeclampsia and Its Subtypes: A Population-Based Study in Beijing, China. Matern-Fetal Med. 2021 Apr;3(2):91.

2.	Young MF, Oaks BM, Tandon S, Martorell R, Dewey KG, Wendt AS. Maternal hemoglobin concentrations across pregnancy and maternal and child health: a systematic review and meta-analysis. Ann N Y Acad Sci. 2019 Aug;1450(1):47–68.

3.	Liu D, Li S, Zhang B, Kang Y, Cheng Y, Zeng L, et al. Maternal Hemoglobin Concentrations and Birth Weight, Low Birth Weight (LBW), and Small for Gestational Age (SGA): Findings from a Prospective Study in Northwest China. Nutrients. 2022 Feb 18;14(4):858.
