# Supplementary material for: Relationships between hemoglobin levels at admission and adverse maternal and perinatal outcomes in patients with preeclampsia
Source: PLoS One. 2025 Oct 16;20(10):e0335079. doi: 10.1371/journal.pone.0335079 (PMC12530512; doi:10.1371/journal.pone.0335079)
Supplement: Attachment — Submitted filename: Response_to_Reviewers_auresp_2.docx [file pone.0335079.s002.docx]

PONE-D-24-46963R1

Relationships between hemoglobin levels at admission and adverse maternal and perinatal outcomes in preeclampsia patients: A retrospective study

PLOS ONE

Dear Dr. He,

Thank you for submitting your manuscript to PLOS ONE. After careful consideration, we feel that it has merit but does not fully meet PLOS ONE’s publication criteria as it currently stands. Therefore, we invite you to submit a revised version of the manuscript that addresses the points raised during the review process.

Please submit your revised manuscript by Nov 10 2025 11:59PM. If you will need more time than this to complete your revisions, please reply to this message or contact the journal office at plosone@plos.org. When you're ready to submit your revision, log on to https://www.editorialmanager.com/pone/ and select the 'Submissions Needing Revision' folder to locate your manuscript file.

Please include the following items when submitting your revised manuscript:

A rebuttal letter that responds to each point raised by the academic editor and reviewer(s). You should upload this letter as a separate file labeled 'Response to Reviewers'.

A marked-up copy of your manuscript that highlights changes made to the original version. You should upload this as a separate file labeled 'Revised Manuscript with Track Changes'.

An unmarked version of your revised paper without tracked changes. You should upload this as a separate file labeled 'Manuscript'.

If you would like to make changes to your financial disclosure, please include your updated statement in your cover letter. Guidelines for resubmitting your figure files are available below the reviewer comments at the end of this letter.

If applicable, we recommend that you deposit your laboratory protocols in protocols.io to enhance the reproducibility of your results. Protocols.io assigns your protocol its own identifier (DOI) so that it can be cited independently in the future. For instructions see: https://journals.plos.org/plosone/s/submission-guidelines#loc-laboratory-protocols. Additionally, PLOS ONE offers an option for publishing peer-reviewed Lab Protocol articles, which describe protocols hosted on protocols.io. Read more information on sharing protocols at https://plos.org/protocols?utm_medium=editorial-email&utm_source=authorletters&utm_campaign=protocols.

We look forward to receiving your revised manuscript.

Kind regards,

Ali Cetin

Academic Editor

PLOS ONE

**Response to Reviewers’ Comments**

Manuscript ID: PONE-D-24-46963R1

Title: Relationships between hemoglobin levels at admission and adverse maternal and perinatal outcomes in patients with preeclampsia

Journal: PLOS ONE

Corresponding Author: Tongqiang He, E-mail: xbfymicu@163.com

Dear Dr. Ali Cetin,

We greatly appreciate your affirmation of our work revising the manuscript, as well as the detailed suggestions you provided for its improvement. According to the requirements in your letter, we have revised the manuscript point by point, as detailed below.

To easily distinguish my answers from editor's comments, we highlighted all our answers in blue while keeping the comments of your letter in black in the Response letter.

Thank you again for your time and consideration.

Looking forward to hearing from you.

Best regards,

Tongqiang He

Department of Obstetrics and Gynecology Intensive Care Unit, Northwest Women's and Children's Hospital

Email: xbfymicu@163.com

Journal Requirements:

If the reviewer comments include a recommendation to cite specific previously published works, please review and evaluate these publications to determine whether they are relevant and should be cited. There is no requirement to cite these works unless the editor has indicated otherwise.

Reply: The reviewers did not request the citation of specific previously published works.

Please review your reference list to ensure that it is complete and correct. If you have cited papers that have been retracted, please include the rationale for doing so in the manuscript text, or remove these references and replace them with relevant current references. Any changes to the reference list should be mentioned in the rebuttal letter that accompanies your revised manuscript. If you need to cite a retracted article, indicate the article’s retracted status in the References list and also include a citation and full reference for the retraction notice.

Reply: We re-retrieved the references cited in the manuscript via PubMed and removed citations of two articles due to incomplete bibliographic information. The relevant details were indicated in the revised manuscript with track changes.

Additional Editor Comments:

In the present form, the manuscript is considerably improved but not finalized. I would like to give you a warning, do not think that it is enough for your article to be corrected by Editing Firms, after all, these people are mostly not obstetricians, in obstetrics, a correct intervention or a correct stitch has a life-saving importance.

Reply: Thank you very much for your reminder. We fully recognize that professional English editing services can help non-native English-speaking researchers clearly convey their research ideas, but they are not the deciding factor in whether a manuscript is published. The scientific content of the research is the main reason for acceptance. Thank you again for your detailed guidance on our manuscript. Following your suggestions, we have carefully revised the manuscript.

Finalize your article by examining the following details related to your article.

\# Comprehensive Manuscript Corrections

\## "Relationships between hemoglobin levels at admission and adverse maternal and perinatal outcomes in preeclampsia patients"

Reply: We have chosen to use "patients with preeclampsia" throughout the manuscript. Therefore, we revised the manuscript's title to "Relationships between hemoglobin levels at admission and adverse maternal and perinatal outcomes in patients with preeclampsia".

---

\## **1. CRITICAL CORRECTIONS - Missing Decimal Points**

\### Abstract Section:

- **Line with SGA**: "SGA(OR: 731; 95% CI: 0.517--1.032)" → **"SGA (OR: 0.731; 95% CI: 0.517-1.032)"**

- **Line with NICU**: "NICU admission(OR: 737; 95% CI: 0.567--0.960)" → **"NICU admission (OR: 0.737; 95% CI: 0.567-0.960)"**

Reply: First, we would like to sincerely apologize for the oversight. We have corrected the two instances of missing decimal points in the abstract section of the manuscript.

---

\## **2. GROUP NAMING STANDARDIZATION**

\### Throughout the manuscript, replace:

- "anemia group" → **"Low Hb group"**

- "normal hemoglobin level group" → **"Normal Hb group"**

- "high hemoglobin level group" → **"High Hb group"**

\### Alternative (if preferred):

- **Group A (anemia)** - hemoglobin ≤109 g/L

- **Group N (normal)** - hemoglobin 110-129 g/L

- **Group H (high)** - hemoglobin ≥130 g/L

Reply: Throughout the manuscript, the terms "anemia group," "normal hemoglobin level group," and "high hemoglobin level group" were replaced with "Low Hb group", "Normal Hb group", and "High Hb group".

---

\## **3. SPACING CORRECTIONS - Add Space Before All Parentheses**

\### Abstract:

- "infants(1--3)" → **"infants (1-3)"**

- "China(4)" → **"China (4)"**

- "infections(5,6)" → **"infections (5, 6)"**

- "age (SGA)(7--9)" → **"age (SGA) (7-9)"**

- "pregnancies(10,11)" → **"pregnancies (10, 11)"**

- "outcomes(12--14)" → **"outcomes (12-14)"**

- "changes(15)" → **"changes (15)"**

\### Methods:

- "tests(creatinine ≥90 µmol/L)" → **"tests (creatinine ≥90 µmol/L)"**

- "dysfunction(NICU)" → **"dysfunction (NICU)"**

- "SGA(defined as" → **"SGA (defined as"**

- "delivery(defined as" → **"delivery (defined as"**

- "delivery(delivery before" → **"delivery (delivery before"**

- "unit(NICU)" → **"unit (NICU)"**

\### Discussion:

- "dysfunction(20)" → **"dysfunction (20)"**

- "flow(19)" → **"flow (19)"**

- "anemia(21--23)" → **"anemia (21-23)"**

- "dysfunction(24--26)" → **"dysfunction (24-26)"**

- "dysfunction(27)" → **"dysfunction (27)"**

- "anemia(28)" → **"anemia (28)"**

- "fraction(29)" → **"fraction (29)"**

- All other reference citations need space before parenthesis

Reply: Thank you for your thorough and patient guidance. We have added space before all parentheses in the manuscript.

---

\## **4. ETHICS APPROVAL SECTION REVISION**

\### Current (problematic):

"Our study complied with the Declaration of Helsinki and was approved by the Ethics Committee of Northwest Women's and Children's Hospital. The ethics committee approved the waiving of informed consent for this research, as it was a retrospective study. The approval number was 2024-012. Patient information was anonymous during the analysis process."

\### **Corrected version:**

"This study complied with the Declaration of Helsinki and was approved by the Ethics Committee of Northwest Women's and Children's Hospital (approval number: 2024-012). Informed consent was waived by the ethics committee due to the retrospective nature of the study. All patient information was anonymized during the analysis."

Reply: We have updated the ethics approval to the correct version in the manuscript.

---

\## **5. TABLE 1 CORRECTIONS**

1. "Severe preeclampsia, n(%)" → **"Severe preeclampsia, n (%)"**

2. "Nulliparas, n(%)" → **"Nulliparas, n (%)"**

3. "Assisted reproduction, n(%)" → **"Assisted reproduction, n (%)"**

Reply: We have added space before all parentheses in table 1.

4. All values: "31.17±4.63" → **"31.17 ± 4.63"** (add spaces around ±)

Reply: We have added spaces around all "±" in table 1.

5. Statistics: "*F*=*0.419*" → **"F = 0.419"** (remove italics from equals sign)

Reply: We have removed italics from equal signs and added spaces around equal signs in Table 1. Only the italicized format of "*F*" has been retained.

6. "*χ^2^*=*6.744*" → **"χ² = 6.744"** (proper chi-square symbol)

Reply: We have corrected the format of the chi-square symbol and added spaces around equal signs.

\### Column Headers:

- "Anemia group" → **"Low Hb group (n=395)"**

- "Normal hemoglobin level group" → **"Normal Hb group (n=877)"**

- "High hemoglobin level group" → **"High Hb group (n=443)"**

Reply: We have revised the column headers of table 1 according to your comments.

---

\## **6. TABLE 2 CORRECTIONS**

1. "Rate,%(95% CI)" → **"Rate, % (95% CI)"**

2. All CI values need spaces: "2.0(0.9,4.0)" → **"2.0 (0.9, 4.0)"**

3. "Post hoc power,%" → **"Post hoc power, %"**

Reply: Thanks for your careful guidance. We have added spaces in the appropriate locations as per your comments.

4. Apply same column header changes as Table 1

Reply: We have modified the column headers in Table 2 as requested.

---

\## **7. STATISTICAL NOTATION STANDARDIZATION**

Throughout manuscript:

- "P value<0.05" → **"P < 0.05"**

- "P values>0.05" → **"P > 0.05"**

Reply: All P values in the manuscript have been modified to P.

- "(OR:3.800; 95% CI:1.677--8.610)" → **"(OR: 3.800; 95% CI: 1.677-8.610)"**

- "(OR:2.224; 95% CI:0.910--5.432)" → **"(OR: 2.224; 95% CI: 0.910-5.432)"**

- "(OR:2.451; 95% CI:1.140--5.268)" → **"(OR: 2.451; 95% CI: 1.140-5.268)"**

Reply: We have made the requested modifications throughout the manuscript.

---

\## **8. RESULTS SECTION CORRECTIONS**

\### Paragraph 1:

- "120.27 g/L(SD 15.52)" → **"120.27 g/L (SD 15.52)"**

- "33.98 weeks(SD 4.18)" → **"33.98 weeks (SD 4.18)"**

Reply: We have added space before parentheses in paragraph 1 of the results section.

\### Paragraph 2:

- "The study population included 395 patients with anemia, 877 patients with normal hemoglobin levels, and 443 patients with high hemoglobin levels."

→ **"The study population comprised 395 patients in the Low Hb group, 877 in the Normal Hb group, and 443 in the High Hb group."**

Reply: We have made the requested modification in paragraph 2 of the results section.

---

\## **9. DISCUSSION SECTION STYLE IMPROVEMENTS**

1. "A series of studies have demonstrated" → **"Several studies have demonstrated"**

2. "Interestingly, our study revealed" → **"Notably, our study revealed"**

3. "These findings contradict the results" → **"These findings contrast with the results"**

4. "Owing to" → **"Due to"** (throughout)

5. "data concerning" → **"data regarding"**

6. "comparatively little research has been devoted" → **"relatively little research has been conducted"**

Reply: Thanks for your guidance. We have made the requested modifications throughout the manuscript.

---

\## **10. REFERENCES FORMATTING**

\### Standardize all references:

1. Month names: Choose either abbreviated (Jun, Sept, Nov) or full (June, September, November) - be consistent

Reply: We uniformly adopt the abbreviated format for months in the reference section.

2. Page ranges: Use en-dash (–) not hyphen (-): "47--68" → **"47–68"**

3. Add spaces: "2019 Aug;1450(1):47--68" → **"2019 Aug; 1450(1): 47–68"**

4. Ensure consistent punctuation after author initials

Reply: We have carefully reviewed the reference formatting and corrected some errors as per your comments.

---

\## **11. TERMINOLOGY CONSISTENCY**

Choose one and use throughout:

- "preeclampsia patients" OR **"patients with preeclampsia"** (preferred)

Reply: We have chosen to use "patients with preeclampsia" throughout the manuscript.

- "hemoglobin concentration" OR **"hemoglobin level"** (choose one)

Reply: We have chosen to use "hemoglobin level" throughout the manuscript.

- "pre-pregnancy BMI" OR **"prepregnancy BMI"** (choose one)

Reply: We have chosen to use "pre-pregnancy BMI" throughout the manuscript.

- "post-partum" → **"postpartum"** (no hyphen)

Reply: We removed the hyphen in "post-partum".

---

\## **12. NUMBER FORMATTING**

- Range dashes: "12--14" → **"12-14"** (use en-dash consistently)

- "3%--5%" → **"3%-5%"**

Reply: We have replaced all hyphens indicating ranges with en-dashes throughout the manuscript.

- Ensure leading zeros: ".731" → **"0.731"**

Reply: We have corrected the two instances of missing zeros and decimal points in the abstract section of the manuscript.

---

\## **13. ABBREVIATION ISSUES TO FIX**

1. **ANOVA** - define at first use

Reply: Since ANOVA appeared only once in the statistical methods section, we removed the abbreviation of ANOVA and retained only the full term “analysis of variance”.

2. Remove redundant definitions of:

- BMI (defined multiple times)

- NICU (defined multiple times)

- SGA (defined multiple times)

- HELLP syndrome (defined multiple times)

Reply: We carefully examined the manuscript and found that these redundant definitions of abbreviations likely occurred because we initially placed table footnotes within the main text. During revisions, we relocated the footnote content to the tables themselves.

---

\## **14. MINOR BUT IMPORTANT CORRECTIONS**

1. Methods section: Add period after "control" in Abstract Methods

Reply: We added a period after "control" in Abstract Methods.

2. "24-hour urine protein" → **"24-h urine protein"** (for consistency)

3. ">40 IU/L" → **"> 40 IU/L"** (add space after >)

4. "≥90 µmol/L" → **"≥ 90 µmol/L"** (add space after ≥)

Reply: We have made the requested modifications in the manuscript.

---

\## **SUMMARY OF KEY CHANGES**

1. **Add spaces** before ALL parentheses

2. **Fix missing decimal points** (0.731, 0.737)

3. **Standardize group names** (Low Hb, Normal Hb, High Hb)

4. **Revise Ethics section** to eliminate redundancy

5. **Fix table formatting** (spaces, statistical notation)

6. **Standardize statistical reporting** (OR: X.XXX; 95% CI: X.XXX-X.XXX)

7. **Choose consistent terminology** throughout

8. **Fix reference formatting** for journal requirements

9. **Under each table, write the definitions of all abbreviations in that table separately.

These corrections will ensure the manuscript meets Q1 journal publication standards.

Reply: Thank you once again for your detailed guidance, which has been invaluable in enhancing our manuscript. We have made the requested revisions in full accordance with your instructions.

[Note: HTML markup is below. Please do not edit.]

While revising your submission, please upload your figure files to the Preflight Analysis and Conversion Engine (PACE) digital diagnostic tool, https://pacev2.apexcovantage.com/. PACE helps ensure that figures meet PLOS requirements. To use PACE, you must first register as a user. Registration is free. Then, login and navigate to the UPLOAD tab, where you will find detailed instructions on how to use the tool. If you encounter any issues or have any questions when using PACE, please email PLOS at figures@plos.org. Please note that Supporting Information files do not need this step.

Reply: All our figures have been checked by PACE.
